# Insights into the autecology of *Aedes albopictus*

**DOI:** 10.1186/s13071-025-07032-2

**Published:** 2025-11-04

**Authors:** Xiang Guo, Ziyao Li, Siyu Zhao, Yijia Guo, Shu Zeng, Haiyang Chen, Xiaohua Liu, Qing He, Liu Ge, Xiaohong Zhou

**Affiliations:** 1 Institute of Tropical Medicine, Department of Pathogen Biology, School of Public Health, Southern Medical University, Guangdong Provincial Key Laboratory of Tropical Disease Research, Key Laboratory of Prevention and Control for Emerging Infectious Diseases of Guangdong Higher Institutes, Key Laboratory of Infectious Diseases Research in South China, Ministry of Education, Guangzhou, 510515 Guangdong China; 2 School of Basic Medical Sciences, Henan University, Kaifeng, China; 3 Department of Laboratory Medicine, The First Hospital of Changsha, (The Affiliated Changsha Hospital of Xiangya School of Medicine, Central South University), Changsha, China; 4 Public Health Service Center, Bao’an District, Shenzhen, China

## Abstract

*Aedes albopictus* is among the 100 most invasive species worldwide and has colonized all continents except Antarctica within the last 30–40 years. As an important vector, it can transmit multiple arboviruses including dengue virus (DENV), Zika virus (ZIKV), and chikungunya virus (CHIKV). Research aimed at understanding the fundamental autecology of this species is essential for advancing the risk of mosquito-borne disease (MBD) and for formulating practical control strategies. This primer provides contextual background information on several key ecological behaviours of *Ae. albopictus*, such as host-seeking and habitat-seeking, from an autecological perspective. This study provides the recent multidisciplinary insights into the ecological behaviours of *Ae. albopictus* and elucidates the connections between its autecology and effective surveillance, disease transmission estimation and efficient disease control.

**Graphical Abstract:**

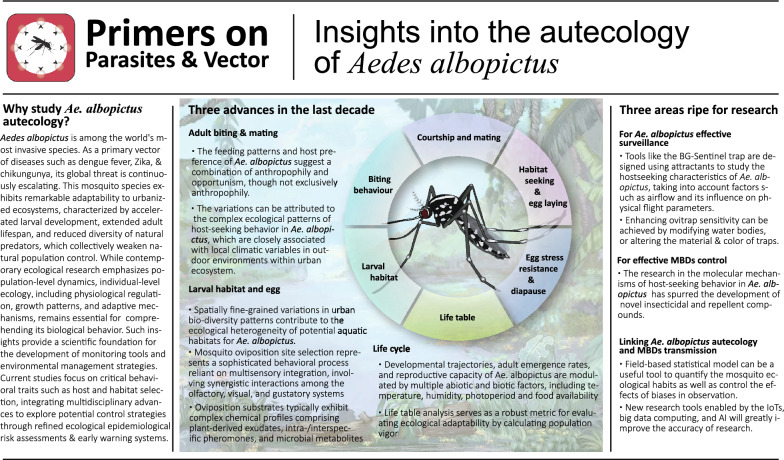

**Supplementary Information:**

The online version contains supplementary material available at 10.1186/s13071-025-07032-2.

## Autecology of *Aedes albopictus*

*Aedes albopictus* ranks among the top 100 invasive species globally, with its range persistently expanding at the periphery of its current distribution [1]. This mosquito species is native to the tropical islands of Southeast Asia in the Western Pacific and the Indian Ocean. It has also successfully invaded and established itself in temperate, subtropical, and tropical regions across all continents except Antarctica [2, 3]. As a key vector for several globally significant diseases, *Ae. albopictus* can transmit dengue virus (DENV), Zika virus (ZIKV) and chikungunya virus (CHIKV) [4–9]. Approximately 390 million DENV infections are estimated to occur annually worldwide [10]. From 1990 to 2021, both the projected age-standardized incidence rate (ASIR) and the age-standardized prevalence rate (ASPR) increased [11, 12]. By 2035, for dengue, the ASIR is estimated to reach 862.23 per 100,000 population, the ASPR is estimated to be 51.60 per 100,000 population, and the age-standardized mortality rate (ASMR) is anticipated to be 0.43 per 100,000 population [12].

Among the diverse mosquito species inhabiting natural environments, *Ae. albopictus* has demonstrated exceptional adaptability to urban ecosystems [13]. This species thrives in urban settings, where its larval development rate is notably accelerated, and its adult survival duration is substantially prolonged [14]. The pronounced reduction in the diversity and numerical abundance of natural predators targeting *Ae. albopictus*, when coupled with the urbanization-induced loss of mosquito interspecies diversity, collectively diminish the efficacy of natural regulatory processes controlling its population density.

**Fig. 1:**
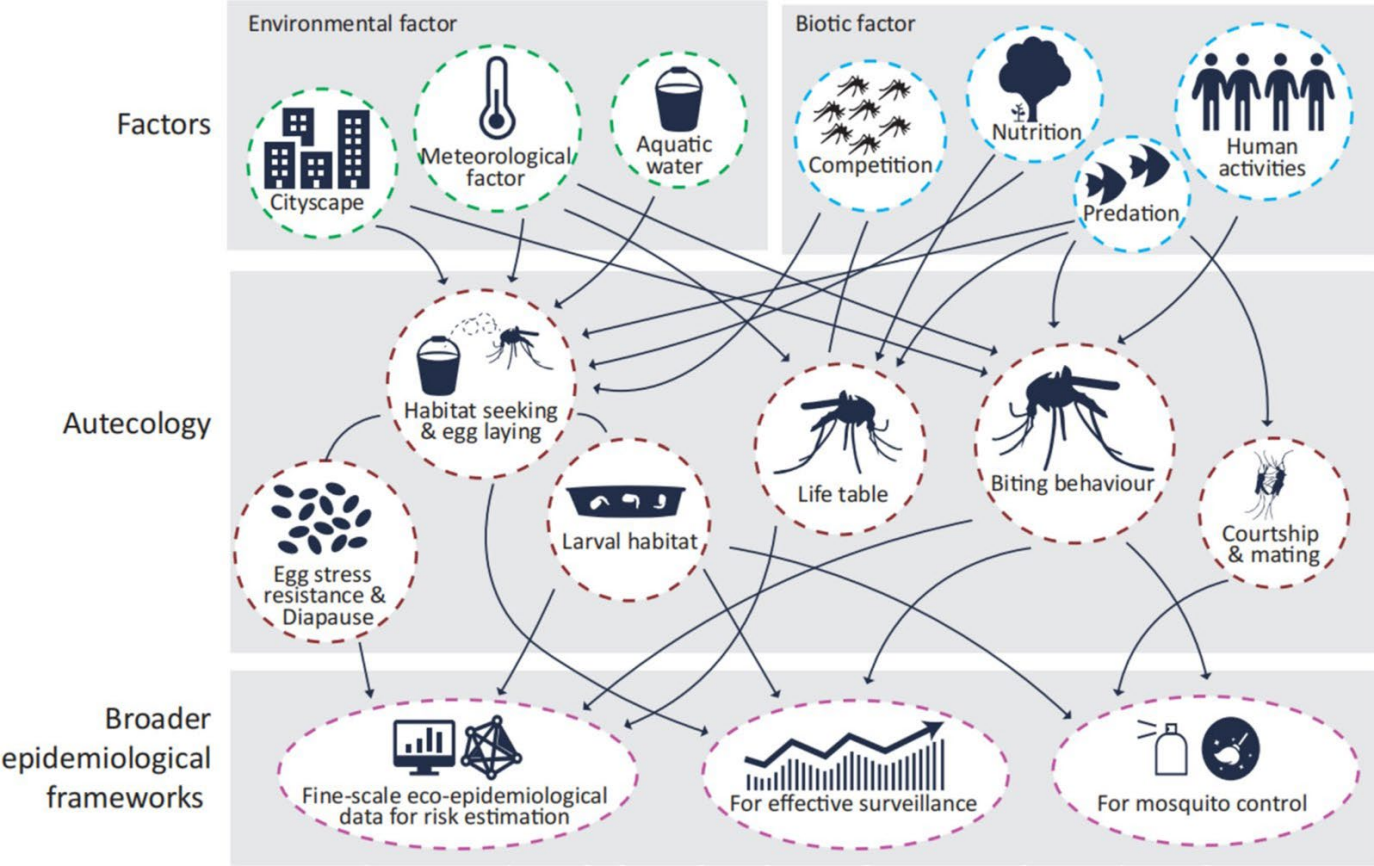
Insights into the autecology of *Aedes albopictus*

Autecology, initially defined as the study of the relationships between individual organisms and their environmental factors, focuses on the ecological adaptations of individual organisms to specific environmental conditions [15]. This encompasses their physiological regulation, growth, and development, as well as other adaptive strategies [15]. Although this concept has receded somewhat in prominence within contemporary ecology because of the prevailing emphasis on population ecology, a nuanced understanding of individual ecology remains pertinent and significant, particularly in the context of mosquito species that serve as vectors for mosquito-borne diseases (MBDs), such as *Ae. albopictus*. In addition to the aforementioned alterations in the ecological behaviour of mosquito vectors within urban environments, the development of monitoring tools for *Ae. albopictus* and the implementation of environmental control measures rely heavily on a comprehensive understanding of its biological habits. With respect to this primer, we attempt to contextualize the ecological habits of *Ae. albopictus* within broader epidemiological frameworks and explore their implications for fine-scale eco-epidemiological risk estimation and early warning systems, along with potential avenues for implementation (Fig. 1).

## The autecology of *Ae. albopictus*: three advances in the last decade

### Adult biting and mating

The biting behaviour of female *Ae. albopictus* is crucially important for MBD transmission [16]. Approximately 30 h post-emergence, adult female *Ae. albopictus* mosquitoes commence biting and blood-feeding [17]. Upon landing on the skin, they swiftly locate an appropriate site and initiate biting. The entire process, from the insertion of mouthparts into the skin to complete abdominal engorgement, typically spans 20–30 s [17]. The volume of blood ingested can range from 0.59–1.37 times its body weight [17, 18]. Additionally, *Ae. albopictus* exhibits a propensity for repeated blood-feeding, with the frequency of refeeding increasing as the interval time increases. Notably, the rate of second blood meals reaches 100% on both the sixth and seventh days following the initial blood meal [18].

The feeding patterns and host preferences of *Ae. albopictus* suggest a combination of anthropophily and opportunism, although not exclusively anthropophily [19]. A literature review revealed that *Ae. albopictus* has a broader host range than *Ae. aegypti* does and feeds on a diverse array of vertebrates [20]. The host-seeking behaviour of *Aedes* mosquitoes is influenced by multiple cues, including visual contrast, chemical volatiles, humidity, and body heat [21–25]. For example, acetophenone, a volatile compound predominantly produced by skin microbiota, is enriched in the volatiles emitted by infected hosts, thereby potently stimulating mosquito olfaction and enhancing attractiveness [26].

In contrast to *Ae. aegypti*, which prefer to rest indoors, *Ae. albopictus* exhibits a preference for outdoor feeding and resting [27, 28]. During favourable periods, the biting behaviour of female mosquitoes in Guangzhou, China, is estimated to occur frequently throughout the day, with a bimodal distribution with peaks occurring within a window of 2–3 h around both dawn and dusk (05:00–08:00 and 16:00–19:00) [29]. Muhammad et al. also reported distinct bimodal biting activity on Penang Island, Malaysia [30]. Conversely, a study conducted in North America revealed that *Ae. albopictus* exhibited high activity during July and September at solar noon [31]. These variations can be attributed to the complex ecological patterns of host-seeking behaviour in *Ae. albopictus*, which are closely associated with local climatic variables in outdoor environments within urban ecosystems [29–32]. According to the ecological model of Yin et al., predicted hourly mosquito densities exhibit non-linear relationships with temperature and illuminance, with density increasing with increasing relative humidity but generally decreasing with increasing wind speed [29]. The estimated temperature range for female biting is 16.4–37.1 °C, with a peak at 26.5 °C [29].

The courtship and mating behaviours of *Ae. albopictus* are characterized as follows: male mosquitoes pursue females through frontal, rear, or lateral approaches [33]. Successful copulations are preceded by longer courtship durations (39 ± 3 s) than unsuccessful attempts (20 ± 2 s) or male rejection behaviour (22 ± 2 s) [33]. Copulation ensues only when the female permits genital contact [33]. During mating, the male’s claspers grasp the female to prevent disengagement, with successful copulations lasting 63 ± 4 s [33]. Post-copulation, males remain adjacent to females for 7 ± 0.4 s before departing [33].

In nature, many dipteran species form aerial swarms containing tens to thousands of males as a prerequisite for mating [34]. However, the swarming behaviour of *Ae. albopictus* has been extensively documented. Instead, urban field studies have revealed a transient correlation between the hourly male mating activity and female host-seeking behaviour [29]. Limited evidence further suggests that photoperiod influences male mating behaviour and reproductive success [29, 33].

Investigations in the southeastern United States, southern China, and Bermuda have revealed an intriguing pattern of asymmetric interspecific mating between *Ae. albopictus* and *Ae. aegypti* [35–38]. Specifically, interspecific matings involving *Ae. albopictus* males and *Ae. aegypti* females occur significantly more frequently than the reciprocal course (*Ae. aegypti* males × *Ae. albopictus* females) [35]. These observed mating biases could potentially enhance the effectiveness of sterile insect technique (SIT) programs aimed at modifying pathogen transmission dynamics in regions where these sympatric species share vectorial capacity for the same pathogens.

### Larval habitat and eggs

The type compositions of *Ae. albopictus* larval habitats are shaped by female adult oviposition preferences. Spatially fine-grained variations in urban biodiversity patterns contribute to the ecological heterogeneity of potential aquatic habitats for this species [39, 40]. In Guangzhou, China, container-type habitats dominate, with plastic containers, metal containers, and ceramic vessels exhibiting the highest habitat preferences, whereas some non-container-type habitats, particularly ornamental ponds and surface water, unexpectedly show moderate colonization rates [41]. In Kurunegala District, Sri Lanka, discarded non-degradable items represent the most prevalent container type, with tyres being the most favourable breeding site [42]. *Aedes albopictus* has also adapted to complex urban environments, including sewers and construction sites [43–45], resulting in highly diverse aquatic habitats.

 Both abiotic and biotic environmental factors directly or indirectly modulate larval development, population density, and spatial distribution. Unsuitable physicochemical conditions, including water temperature, water pH, inorganic salt concentrations, and dissolved oxygen levels, exert toxic effects on larvae [46, 47]. For example, the mortality rates of third- and fourth-instar larvae reached 83.8 ± 8.7% in 1.0% NaCl solution and 100% in 3.0% NaCl solutions [47]. In addition to posing predation threats from natural enemies such as *Toxorhynchites splendens*, *Ae. albopictus* larvae compete with conspecific and heterospecific mosquito larvae for survival [48]. Moreover, carryover effects from larval exposure to environmental bacteria influence adult phenotypic traits. Colonization by different native bacterial isolates during larval development significantly affects pupation rates and adult body size but not longevity [49]. Larval exposure to an *Enterobacteriaceae* isolate reduced antibacterial activity in adult haemolymph and decreased DENV dissemination titres [49].

 Female mosquitoes, which are replete with mature eggs, actively seek suitable aquatic habitats for oviposition. Ideal breeding sites provide abundant food resources while minimizing predation risk. Different mosquito species exhibit distinct habitat preferences: *Aedes* spp. favour container-type microhabitats, *Culex* spp. predominantly colonize eutrophic stagnant waters, and *Anopheles* spp. preferentially select large-scale rice field ecosystems [50–52]. For *Ae. albopictus*, gravid females demonstrate pronounced preferences for dark-coloured containers with low illuminance and rough surfaces [53, 54].

Mosquito oviposition site selection represents a sophisticated behavioural process that relies on multisensory integration and involves synergistic interactions among the olfactory, visual, and gustatory systems [55–57]. The olfactory system is particularly well characterized, with molecular studies demonstrating that the olfactory co-receptor Orco regulates oviposition preference in gravid *Anopheles sinensis* and correlates with reproductive fitness in *Ae. aegypti* [58, 59]. In *Ae. albopictus*, odorant-binding proteins (OBPs), which are critical functional molecules in olfactory recognition pathways, have been mechanistically validated [60]. Specifically, the *Ae. albopictus* *OBP67* and *OBP56d*-like genes mediate oviposition site localization [60].

 The visual system of *Ae. albopictus* deciphers environmental optical signals via its compound eyes, each composed of ommatidia containing eight photoreceptor cells. Spectral sensitivity is determined by opsin-mediated phototransduction mechanisms [61, 62]. Functional genomic analyses have revealed that the *rho-l* and *Kh* genes play critical roles in modulating photic threshold regulation, thereby influencing crepuscular oviposition site-seeking behaviours [63].

 Oviposition substrates typically exhibit complex chemical profiles comprising plant-derived exudates, intra-/interspecific pheromones, and microbial metabolites [41, 64–68]. Close-range substrate evaluation involves multisensory integration: in addition to olfactory cues, tarsal-specific gustatory receptors (e.g., the *GR11* gene in *Ae. albopictus*) facilitate contact-mediated chemoperception for identifying aquatic sites [69, 70]. Notably, systematic mapping of chemical ligands to their cognate receptors remains incomplete. Future research should integrate metabolomic-receptomic approaches with field-based ecological validations to further elucidate the chemical ecology underlying mosquito oviposition strategies.

Desiccation and low temperature represent critical environmental stressors affecting the survival of *Ae. albopictus*. Newly oviposited eggs lack desiccation resistance because of underdeveloped protective structures; however, at 11–13 h post-oviposition, embryos with fully developed serosal cuticles can remain viable under dry conditions for several days [71]. At 25 °C and 92% relative humidity (RH), hatch rates remain high (75%) even after 4 months of storage [17]. Conversely, decreasing humidity levels progressively reduce hatching success: after 5 days at 25 °C, hatch rates reach 90.5% at 75% RH but decline to 65%, 40%, and 3% at 50%, 3%, and 0% RH, respectively [17].

With respect to cold tolerance, *Ae. albopictus* eggs can survive at temperatures as low as −5 °C for 30 days, whereas tropical strains tolerate −10 °C for 2 days, subtropical strains for 10 days, and temperate strains for 20 days [72]. Eggs exposed to −15 °C fail to hatch [73].

 Diapause, a preprogrammed dormancy strategy in mosquitoes, persists despite favourable environmental conditions [74]. When adult females of *Ae. albopictus* experience short day lengths, they produce diapause eggs that remain unhatched but arrest development at the pharate first-instar larval stage [75]. Compared with non-diapause eggs, diapause eggs exhibit developmental arrest, reduced metabolic activity, increased desiccation resistance, and decreased cold tolerance [73, 76]. For example, European *Ae. albopictus* diapause eggs tolerate −10 °C during prolonged exposure (12–24 h) and −12 °C for 1 h [73]. Diapause enables survival in unfavourable environments, such as in winter conditions or suboptimal habitats, significantly increasing the adaptability of the species across diverse climatic and geographical regions. The first well-established *Ae. albopictus* population in Texas, USA (summer 1985), likely originated from imported car tyres containing dormant eggs from Asia [77].

### Life table

Through systematic observation of the entire developmental process of *Ae. albopictus*, key demographic parameters, including stage-specific development duration, survival rates, age-group proportions, mean generation time, and intrinsic rate of increase, can be accurately calculated. In a laboratory-based life table study conducted at 28.6 °C and RH of 64%, the total pre-adult development time averaged 9.47 days, with stage-specific durations as follows: 1.48 ± 0.05 days for eggs, 1.79 ± 0.04 for first instars, 1.35 ± 0.05 for second instars, 1.31 ± 0.05 for third instars, 1.55 ± 0.06 for fourth instars, and 1.99 ± 0.02 for pupae. Adult longevity differed significantly between the sexes, with females surviving 23.78 ± 0.69 days and males surviving 19.43 ± 0.55 days [78].

The developmental trajectories, adult emergence rates, and reproductive capacity of *Ae. albopictus* are modulated by multiple abiotic and biotic factors, including temperature, humidity, photoperiod, and food availability [79, 80]. In semi-field or field environments, stage-specific development times and survivorship exhibit seasonal variability [79]. Urban ecosystems further act as potent drivers, accelerating mosquito development and increasing survival rates [14].

Life table analysis serves as a robust metric for evaluating ecological adaptability by calculating population vigour through the following formula: egg number × hatch rate × emergence rate * pupation rate * adult survival time * average adult weight * average wing length/(larval development time + pupae development time) [81–83]. For instance, the F1534S mutation, a prevalent knockdown resistance (kdr) variant conferring deltamethrin resistance, imposes significant fitness costs, as evidenced by reduced population vigour metrics [84]. Population structure analyses of laboratory-selected resistant strains revealed that the acquisition of alternative mutations (e.g., I1532T) with lower fitness penalties enables a more favourable trade-off between resistance and adaptability [81, 85].

## The autecology of *Ae. albopictus*: three areas ripe for research

### Advancing effective surveillance of *Ae. albopictus* through a comprehensive understanding of autecology

Given the challenges in directly quantifying mosquito population sizes, ecological behaviour frequencies are often employed as proxies for estimating changes in population density. A thorough understanding of these behavioural patterns enables the development of more effective monitoring strategies: (1) host-seeking behaviour analysis tools, such as the BG-Sentinel trap, leverage olfactory attractants to investigate the host-seeking behaviour of *Ae. albopictus*, incorporating aerodynamic considerations that influence flight dynamics (Table 1) [86, 87]; and (2) ovitrap optimization, in which the sensitivity can be enhanced through modifications to breeding sites, including the addition of chemical attractants or adjustments to trap material composition and colouration (Table 1) [53, 88, 89].

**Table 1:** Summary of the ecological habits of *Aedes albopictus* and their implications for effective surveillance and transmission control

Ecological habits	For effective surveillance	Implication for MBD control	References
Biting behaviour	Development of a trap and trap attractants	Development of repellent Strategy for adulticide applications	[26, 86, 87]
Habitat-seeking and egg-laying	Use and improvement of the ovitrap	Design of lethal ovitrap	[53, 88, 89]
Egg resistance and diapause	–	Photoperiod manipulation strategy of diapause for mosquito control	[90]
Larval habitat	–	Development of larvicide; improvement of the environment and cleaning of habitats	[41, 91]

### Elucidating the autecology of *Ae. albopictus* to optimize MBD control strategies

In the context of MBD transmission dynamics, the molecular mechanisms of host-seeking behaviour in *Ae. albopictus* represent a critical research focus, with scientists actively elucidating its ecological significance [24, 25]. This has spurred the development of novel insecticidal and repellent compounds targeting host-seeking mechanisms. Notably, Zhang et al. demonstrated that dietary supplementation with a vitamin A derivative in virus-infected mice significantly reduced acetophenone-mediated mosquito attraction and subsequent viral transmission [26]. VanderGissen et al. revealed that commercial soaps differentially modulated *Aedes* host selection—some enhancing human attractiveness and others exerting repellent effects—suggesting opportunities for reverse-engineering chemical mixtures to develop synthetic repellent formulations [92].

###  Integrating *Ae. albopictus* autecology with MBD transmission dynamics

 Information integration, digital transformation, and intelligent augmentation constitute the core objectives of developing a global high-efficiency risk estimation and early warning system for MBDs. To construct an eco-epidemiological multi-scale framework for archiving these goals, it is critical to conduct coarse-to-fine-scale investigations into mosquito biology that elucidate the mechanistic linkages among environmental drivers, mosquito life-history traits, and MBD transmission dynamics [93]. Field-based epidemiological studies inherently confront unpredictable biases arising from environmental variability, observer subjectivity, and sampling heterogeneity. Statistical models grounded in ecological field data serve as powerful analytical tools for quantifying mosquito behavioural ecology while controlling for observational biases. The advent of Internet of Things (IoT)-enabled sensors, big data analytics, and artificial intelligence (AI) algorithms promises to significantly reduce methodological complexities and enhance research precision in future MBD surveillance systems. However, this technological integration necessitates expanded interdisciplinary collaboration, requiring the recruitment of professionals with specialized expertise in mechanical engineering, computational statistics, and software architecture to augment existing mosquito ecology research teams.

##  Conclusions

*Aedes albopictus* is an important vector for transmitting several pathogens, such as DENV, worldwide. In this primer, we presented the complex ecological habits of *Ae. albopictus*. Recent advances indicate that exploring the ecological habits of *Ae. albopictus* requires multidisciplinary methods. The results of these multidisciplinary capabilities will strongly support the development of drugs, repellents, or other effective *Ae. albopictus* control methods to effectively mitigate MBDs.

## Supplementary Information

Additional file 1. Downloadable poster describing the autecology of *Ae. albopictus.*

## Data Availability

Data supporting the main conclusions of this study are included in the manuscript.
