# Supplementary material for: Insights into the autecology of Aedes albopictus
Source: Parasit Vectors. 2025 Nov 4;18:445. doi: 10.1186/s13071-025-07032-2 (PMC12584224; doi:10.1186/s13071-025-07032-2)
Supplement: Supplementary file 1 — Additional file 1. Downloadable poster describing the autecology of Ae. albopictus. [file 13071_2025_7032_MOESM1_ESM.pdf]

Primers on Parasites & Vector

Insights into the autecology of *Aedes albopictus*

Why study *Ae. albopictus* autecology?

Aedes albopictus is among the world's most invasive species. As a primary vector of diseases such as dengue fever, Zika, & chikungunya, its global threat is continuously escalating. This mosquito species exhibits remarkable adaptability to urbanized ecosystems, characterized by accelerated larval development, extended adult lifespan, and reduced diversity of natural predators, which collectively weaken natural population control. While contemporary ecological research emphasizes population-level dynamics, individual-level ecology, including physiological regulation, growth patterns, and adaptive mechanisms, remains essential for comprehending its biological behavior. Such insights provide a scientific foundation for the development of monitoring tools and environmental management strategies. Current studies focus on critical behavioral traits such as host and habitat selection, integrating multidisciplinary advances to explore potential control strategies through refined ecological epidemiological risk assessments & early warning systems.

Three advances in the last decade

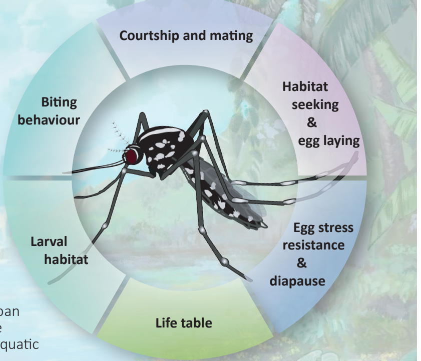

Adult biting & mating

- The feeding patterns and host preference of *Ae. albopictus* suggest a combination of anthropophily and opportunism, though not exclusively anthropophily.
- The variations can be attributed to the complex ecological patterns of host-seeking behavior in *Ae. albopictus*, which are closely associated with local climatic variables in outdoor environments within urban ecosystem.

Larval habitat and egg

- Spatially fine-grained variations in urban bio-diversity patterns contribute to the ecological heterogeneity of potential aquatic habitats for *Ae. albopictus.*
- Mosquito oviposition site selection represents a sophisticated behavioral process reliant on multisensory integration, involving synergistic interactions among the olfactory, visual, and gustatory systems
- Oviposition substrates typically exhibit complex chemical profiles comprising plant-derived exudates, intra-/interspecific pheromones, and microbial metabolites

Life cycle

- Developmental trajectories, adult emergence rates, and reproductive capacity of Ae. albopictus are modulated by multiple abiotic and biotic factors, including temperature, humidity, photoperiod and food availability
- Life table analysis serves as a robust metric for evaluating ecological adaptability by calculating population vigor

Three areas ripe for research

For *Ae. albopictus* effective surveillance

- Tools like the BG-Sentinel trap are designed using attractants to study the hostseeking characteristics of *Ae. albopictus*, taking into account factors such as airflow and its influence on physical flight parameters.
- Enhancing ovitrap sensitivity can be achieved by modifying water bodies, or altering the material & color of traps.

For effective MBDs control

- The research in the molecular mechanisms of host-seeking behavior in *Ae. albopictus* has spurred the development of novel insecticidal and repellent compounds.

Linking *Ae. albopictus* autecology and MBDs transmission

- Field-based statistical model can be a useful tool to quantify the mosquito ecological habits as well as control the effects of biases in observation.
- New research tools enabled by the IoTs, big data computing, and AI will greatly improve the accuracy of research.
